# Complete mitochondrial genome of a potential vector louse fly, *Lipoptena grahami* (Diptera, Hippoboscidae)

**DOI:** 10.1080/23802359.2021.1931508

**Published:** 2021-05-24

**Authors:** Mo Wang, Jishan Wang, Yingya Guo, Qinglian Zheng, Dibo Nouhoum, Fanming Meng

**Affiliations:** a Department of Medical Parasitology, School of Basic Medical Sciences, Central South University, Changsha, China; b Key Laboratory for Conserving Wildlife with Small Populations in Yunnan, Faculty of Biodiversity Conservation, Southwest Forestry University, Kunming, China; c China Forest Exploration & Design Institute in Kunming, National Forestry and Grassland Administration, Kunming, China; d Southwest Monitoring Center of Protected Area and Wildlife, National Forestry Administration, Kunming, China; e Research center of Asian elephant, National Forestry and Grassland Administration, Kunming, China;; f Damdo Forestry and Grassland Bureau, Changdu, China

**Keywords:** Mitogenome, blood-feeding, ectoparasite, wingless, parasitology

## Abstract

*Lipoptena grahami* Bequaert, 1942 (Diptera, Hippoboscidae) was first described in China almost 80 years ago. Species of *Lipoptena* were obligate blooding-feeding insects and commonly reported as vectors of wild animals of Cervinae. The complete mitochondrial genome of *L. grahami* was assembled to 16,953 bp in length. The AT content of *L. grahami* mitogenome is 80.59%. In total, 22 tRNAs, 2 rRNAs, and 13 protein-coding genes (PCGs) were annotated from *L. grahami*’s mitogenome. The typical clover-leaf structure of tRNAs was also analyzed and confirmed except the tRNA-Ser (AGN). A phylogenetic tree was constructed based on *L. grahami* with some other fly species.

Louse flies (Diptera, Hippoboscidae) are obligate blooding-feeding insects. Species of this family are typical ectoparasites of birds or mammals. *Lipoptena* is a large group of Hippoboscidae (Maa 1965, 1969). Species like *L. fortisetosa* and *L. cervi* are globally reported as parasitic vectors of wild animals of Cervinae, and also risks of livestock (Kim et al. 2010; Andreani et al. 2019). *Lipoptena grahami* was first recorded and described in the study of Bequaert in 1942 (Bequaert 1942) in Sichuan Province of China. Since the lack of genetic background and information of louse fly in Hippoboscidae, completed mitochondrial genome of *L. grahami* was sequenced and annotated here.

The specimens were found on a dead ungulate, and collected using forceps by Qinglian Zheng and Yingya Guo at Karuo, Changdu, Tibet Autonomous Region (97°20’E, 31°07’N) in 13 August 2019. These wingless flies which hid under the fur of the dead animal were easy to be captured. The living louse fly samples were restored in 99% ethanol. Species identification was performed in the lab with a microscope using the morphological characters described in the literature (Maa 1965, 1969).

A unique code (CSU-MG20191210-11) of voucher sample was given and deposited in parasitology herbarium of the Department of Medical Parasitology Science, Central South University. The DNA material was extracted from louse fly samples using the CATB method following the previous study (Skevington and Yeates 2000), then purified with QIAQuick PCR Purification Kit (ID: 28106) (Qiagen, Germany). Then DNA material was amplified with several pairs of overlapped PCR primers for further genome construction work. The sequencing was performed on ABI PRISM 3130 platform (Applied Biosystems, Foster, CA). The mitochondrial genome was assembled using GetOrganelle software (v1.7.4-pre2) and annotated roughly following the procedure described before (Jin et al. 2020). First, raw mitogenomic sequences were imported into MITOS web server to determine the approximate boundaries of genes. The exact positions of protein-coding genes (PCGs) were found by searching for ORFs. All tRNAs were identified using ARWEN (Laslett and Canback 2008), DOGMA (Wyman et al. 2004), and MITOS (Bernt et al. 2013). Then the mitogenome of *L. grahami* was submitted into GenBank and got its accession number (MT679542).

The phylogenetic history was constructed using *L. grahami* with other fly species chosen from some families of Calyptratae of Diptera, except the outgroup *Drosophila melanogaster* (Figure 1). The sequence resource of other species was downloaded from the GenBank. Sequences were aligned in Clustal Omega (v1.2.1) (Sievers et al. 2011) with default parameter settings. MEGA5 was used for phylogenetic history analysis with the Maximum-likelihood method, bootstrap = 1000 (Tamura et al. 2011). According to the result, the species of the same family were all cluster into their own branch. The *L. grahami* with *Melophagus ovinus*, another common louse fly species, formed a single clade. Considering the rare of mitochondrial genome resource of Hippoboscidae, present work provided important information for further mitogenomic research on louse fly species.

**Figure 1.**
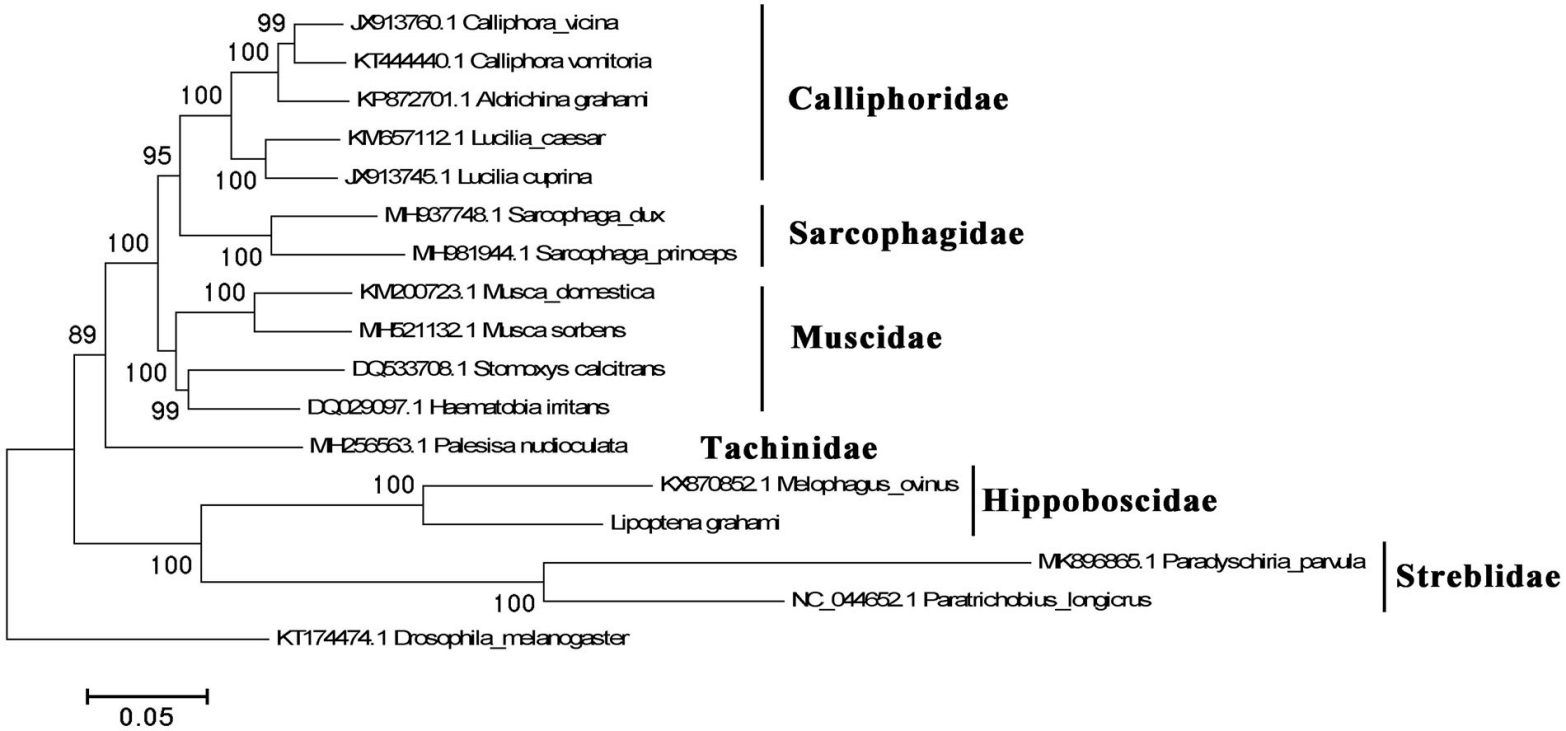
The maximum-likelihood tree was constructed using *L. grahami* and *M. ovinus* with other 15 fly species.

## Data Availability

The data that support the findings of this study are openly available in GenBank at https://www.ncbi.nlm.nih.gov/nuccore/MT679542.
